# Two-Stage Interference Cancellation for Device-to-Device Caching Networks

**DOI:** 10.3390/s20030780

**Published:** 2020-01-31

**Authors:** Sang-Woon Jeon, Sung Ho Chae

**Affiliations:** 1 Department of Military Information Engineering, Hanyang University, Ansan 15588, Korea; sangwoonjeon@hanyang.ac.kr; 2 Department of Electronic Engineering, Kwangwoon University, Seoul 01897, Korea

**Keywords:** caching, cooperative transmission, D2D communication, interference cancellation, Internet of things, outage capacity, sensor networks

## Abstract

Wireless device-to-device (D2D) caching networks are studied, in which *n* nodes are distributed uniformly at random over the network area. Each node caches *M* files from the library of size m≥M and independently requires a file from the library. Each request will be served by cooperative D2D transmission from other nodes having the requested file in their cache memories. In many practical sensor or Internet of things (IoT) networks, there may exist simple sensor or IoT devices that are not able to perform real-time rate and power control based on the reported channel quality information (CQI). Hence, it is assumed that each node transmits a file with a fixed rate and power so that an outage is inevitable. To improve the outage-based throughput, a cache-enabled interference cancellation (IC) technique is proposed for cooperative D2D file delivery which first performs IC, utilizing cached files at each node as side information, and then performs successive IC of strongly interfering files. Numerical simulations demonstrate that the proposed scheme significantly improves the overall throughput and, furthermore, such gain is universally achievable for various caching placement strategies such as random caching and probabilistic caching.

## 1. Introduction

Wireless traffic has grown exponentially in recent years, mainly due to on-demand video streaming and web browsing [1]. To support such soaring traffic, wireless caching has been actively studied as a promising solution to cost-effectively boost the throughput of wireless content delivery networks. Together with the main trend on the fifth-generation (5G) cell densification with different hierarchy consisting of macro, small, and femto base stations (BSs), caching objects have been moved to network edges, called edge-caching [2,3,4].

Wireless caching techniques in general consist of two phases: the *file placement phase* and the *file delivery phase*. Most existing works have studied the joint design of file placement and delivery strategies to optimize the hit probability, network throughput, etc., for examples, see [2,3,4,5,6,7] and the references therein. Although the joint design and optimization of the file placement and delivery schemes can improve the performance of caching networks, it might excessively increase the system complexity and signaling overhead and, furthermore, such optimization requires a priori knowledge of the file popularity profile, which is very challenging for most of the sensor or IoT networks implemented with low-cost hardware devices that have limited communication and computing capabilities. To address such limitations in sensor or IoT devices, in this paper, we rather focus on developing an efficient file delivery scheme that can provide an improved throughput with limited signaling compared to the conventional file delivery schemes and can be universally applied to *any* file placement strategies. In particular, we propose cooperative device-to-device (D2D) file delivery at the transmitter side and cache-enabled interference cancellation (IC) technique, which first performs IC based on cached files and then performs successive IC of strongly interfering files, at the receiver side. By numerical simulations, we show that the proposed scheme can improve the outage-based throughput regardless of the choice of file placement strategies.

To the best of our knowledge, our work is the first attempt to provide a unified IC framework for wireless D2D caching networks incorporating cooperative transmission from multiple nodes sending the same file [4,8], IC by utilizing cached files as side information [6,8], and IC by interference decoding of undesired files [9,10]. (Throughout the paper, we assume that point-to-point Shannon-capacity-achieving channel codes are used to transmit each file. Hence, a transmitted file will be successfully decoded if its received signal-to-interference-plus-noise ratio (SINR) is greater than $2^{R}-1$, where *R* is the transmission rate of the file.) As a result, the proposed scheme attains a synergistic gain that is able to improve the overall throughput of D2D caching networks, universally achievable for any file placement strategies. The key observation is that the same encoded bits induced by the same file request can be transmitted by multiple nodes for D2D caching networks, and these superimposed signals can be treated as a single received signal for decoding at each node. Notice that such a decoding procedure at each node is indeed beneficial for decoding not only the desired file but also interfering files, i.e., undesired files, because it boosts the received power of interfering files so that a subset of strongly interfering files becomes decodable and, as a result, each node can cancel out their interference before decoding the desired file. Furthermore, treating multiple simultaneously transmitted signals as a single aggregate signal drastically reduces the decoding complexity, i.e., the optimization procedure of establishing an optimal subset of interfering files for successive IC as well as their optimal decoding order.

### 1.1. Related Work

Recently, D2D caching has been actively studied, in order to allow end terminals to cache content files and then to directly serve each other via D2D single-hop or multihop file delivery [5,6,7,8,11,12]. Specifically, the optimal throughput scaling laws for D2D caching networks achievable by single-hop file delivery and multihop file delivery were derived in [5,11,12]. Analytical modeling for traffic dynamics has been studied in [6] to investigate packet loss rates for cache-enabled networks. In [8], IC by using cached files as side information at each receiver was proposed for cache-enabled networks. Caching placement optimization for probabilistic random caching was studied in [7].

In addition to improving throughput performance of wireless networks, caching has also been considered as a core technology for enhancing or guaranteeing various quality of service (QoS) requirements in sensor or Internet of things (IoT) applications. To reduce energy consumption of wireless sensor networks, node selection for caching sensor data to reduce the communication cost was studied in [13], and dynamic selection of such nodes was further proposed in [14]. A cache-based transport layer protocol was proposed to reduce end-to-end delivery cost of wireless sensor networks in [15]. A comprehensive survey on the state-of-the-art cache-based transport protocols in wireless sensor networks such as cache insertion/replacement policy, cache size requirement, cache location, cache partition, and cache decision was provided in [16]. Recently, caching strategies to support various IoT applications were studied [17,18,19,20]. It was shown in [17] that in-network caching of IoT data at content routers significantly reduces network load by caching highly requested IoT data. Energy-harvesting-based IoT sensing has been studied in [18], which analyzes the trade-off between energy consumption and caching. In [19], a cooperative caching scheme that is able to utilize resource pooling, content storing, node locating, and other related situations was proposed for computing in IoT networks. In [20], an energy-aware dynamic resource caching strategy to enable a broker to cache popular resources was proposed to maximize the energy savings from servers in smart city applications.

### 1.2. Paper Organization

The rest of the paper is organized as follows. The problem formulation, including the network model and performance metric used in this paper, is described in Section 2. The proposed cache-enabled IC scheme is given in Section 3. The performance evaluation of the proposed scheme is provided in Section 4. Finally, Section 5 concludes the paper.

## 2. Problem Formulation

For notational convenience, [1:n] denotes {1,2,⋯,n} in this paper. We also use the subscript ${\left(\cdot \right)}_{i}$ (without bracket) for node indices and the subscript ${\left(\cdot \right)}_{\left[k\right]}$ (with bracket) for file indices.

### 2.1. Wireless D2D Caching Networks

We consider a wireless D2D network in which *n* nodes are uniformly distributed at random over the network area of size ${\left[0,a\right]}^{2}$ and assumed to operate in half-duplex mode. We assume a static network topology, i.e., once the positions of *n* nodes are given, they remain the same during the entire time block. Let $q_{i}\in {\left[0,a\right]}^{2}$ denote the position vector of node *i* and $d_{ij}$ denote the Euclidean distance between nodes *i* and *j*, where i,j∈[1:n]. That is, $d_{ij}=∥q_{i}-q_{j}∥$, where ∥·∥ denotes the Euclidean norm of a vector. The path-loss channel model is assumed in which the channel coefficient from node *i* to node *j* at time *t* is given by $h_{ji}\left[t\right]=\frac{q_{ji}\left[t\right]}{d_{ji}^{\gamma /2}}$. Here, γ≥2 is the path-loss exponent and $q_{ji}\left[t\right]$ is the short-term fading at time *t*, which is drawn independent and identically distributed (i.i.d.) from a continuous distribution with zero mean and unit variance. The received signal of node *j* at time *t* is given by

(1) $$\begin{array}{c} y_{j}\left[t\right]=\sum_{i=1,i\notin j}^{n}h_{ji}\left[t\right]x_{i}\left[t\right]+n_{j}\left[t\right], \end{array}$$

where $x_{i}\left[t\right]$ is the transmit signal of node *i* and $n_{j}\left[t\right]$ is the additive noise at node *j* that follows CN(0,1). Each node should satisfy the average transmit power constraint, i.e., $E\left(|x_{i}{|}^{2}\left[t\right]\right)\leq P$ for all i∈[1:n]. For notational simplicity, we omit the time index *t* from now on.

As in the general case, a caching scheme consists of two phases: the file placement phase and the file delivery phase. We assume a library $F=\{f_{\left[1\right]},f_{\left[2\right]},\cdots ,f_{\left[m\right]}\}$ consisting of *m* files with equal size. During the file placement phase, each node *i* stores *M* files in its local cache memory from F, where M<m. Let $p_{r}\left(F\right)=\left[p_{r}\left(f_{\left[1\right]}\right),p_{r}\left(f_{\left[2\right]}\right),\cdots ,p_{r}\left(f_{\left[m\right]}\right)\right]$ be the file popularity distribution, where $p_{r}\left(f_{\left[k\right]}\right)$ denotes the demand probability of file $f_{\left[k\right]}$, k∈[1:m]. Without loss of generality, we assume $p_{r}\left(f_{\left[k\right]}\right)\geq p_{r}\left(f_{\left[l\right]}\right)$ if k≤l. During the file delivery phase, each node *i* demands a file in F—independently, according to the file popularity distribution $p_{r}\left(F\right)$—and the network operates in a way to satisfy the requested file demands.

### 2.2. Outage-Based Throughput

Various sensor or IoT networks are currently implemented using low-cost hardware devices with limited communication and computing capabilities [21]. Hence, the joint optimization of the file placement and delivery schemes and implementation of such a centralized optimal solution might be impractical in wireless D2D caching networks. To overcome such limitation, in this paper, we focus on developing an efficient file delivery protocol for wireless D2D caching networks after the file placement phase is completed, i.e., *M* cached files are already stored in each cache memory via an arbitrary file placement strategy. Let $F_{i}\subset F$ denote the *M* cached files stored in the cache memory of node *i*. During the file delivery phase, each node *i* transmits one of the files in $F_{i}$ when it is requested by neighbor nodes, which will be specified in Section 3.

In many practical communication systems, especially for sensor or IoT applications, there may exist simple sensor or IoT devices that are not able to perform real-time rate and power control based on the reported channel quality information (CQI). Therefore, we assume that each node *i* transmits a file with fixed rate *R* and power *P*, i.e., no rate and power adaptation is applied. Then, by applying a point-to-point Shannon-capacity-achieving channel code, the transmitted file can be decoded at the receiver side if the received signal-to-interference-plus-noise ratio (SINR) is greater than $2^{R}-1$. Consequently, an outage occurs for some nodes if their capacity limits, which are determined by their SINR values, are smaller than the transmission rate *R*. The outage-based throughput is then given by *R* times the number of nodes that can successfully decode their desired files, i.e.,

(2) $$\begin{array}{c} .T=R\sum_{i=1}^{n}I\left({\hat{f}}_{i}=f_{i}\right), \end{array}$$

where I(·) is the indicator function of an event, which is one if the event occurs or zero otherwise. Here, $f_{i}$ denotes the desired file at node *i* and ${\hat{f}}_{i}$ denotes the estimated version of it at node *i*. For the rest of the paper, we will propose a cache-enabled IC scheme for improving the outage-based throughput in (2) for a given file placement strategy.

**Remark** **1** *
(File demand in its local cache memory).* *Since each node demands a file according to $p_{r}\left(F\right)$, the desired files of some nodes might be already stored in their cache memories for some cases. Those files can be immediately delivered so that the throughput in (2) defined as bps/Hz becomes infinity if we count such a delivery, because the transmission time is zero for this case. To avoid such trivial cases, we only count the file delivery from other nodes when calculating (2).*

## 3. Cache-Enabled Interference Cancellation

In this section, we propose a cache-enabled IC scheme for cooperative D2D file delivery. In particular, the proposed scheme performs a two-step procedure. The first step is to select a cached file to transfer from each node and then send the selected file cooperatively. The second step is to cancel out interference at each node in order to improve the outage-based throughput, in which each node first performs IC based on its own cached files and then performs successive IC of strongly interfering files. In the following, we describe the first and second steps of the proposed scheme in details.

For ease of explanation, we assume that the desired file of each node is not included in its local cache memory, i.e., $f_{i}\notin F_{i}$, for the rest of this section, as also mentioned in Remark 1.

### 3.1. Step 1: File Request and Transfer at the Transmitter Side

In this subsection, we state how to select a file to transfer for each node *i* among its *M* cached files in $F_{i}$. Let δ>0 denote the maximum allowable distance for single-hop D2D file delivery, which will be numerically optimized later. Node *i* broadcasts a file request message of the desired file $f_{i}$, denoted by ${\tilde{f}}_{i}$ (the file index of $f_{i}$), to the other nodes. This file request procedure can be performed based on a predetermined round-robin manner via extra interactive signals, similarly assumed in [6,8]. Note that node *i* might have multiple requested files or might not have any requested files in its cache memory after finishing the above file request message broadcasting. Then, node *i* first broadcasts a file transmission message of file $f_{i^{*}}$, denoted by ${\tilde{\tilde{f}}}_{i^{*}}$ (again, the file index of $f_{i^{*}}$), if node *i* has $f_{i^{*}}$ in its cache memory and node $i^{*}$ is the closest node of node *i* within the radius of δ among nodes that satisfy $f_{i^{*}}\in F_{i}$, where $i\neq i^{*}$. It is assumed that after the file request transmission, each node can know the distance between itself and the other nodes by utilizing CQI at the receiver side. If there is no such node $i^{*}$ for node *i*, node *i* does not broadcast the file transmission message. As the same manner used for the file request message broadcasting, such procedure can be performed based on a predetermined round-robin manner. It is assumed that the size of ${\tilde{f}}_{i}$ and ${\tilde{\tilde{f}}}_{i}$ (file indices) is much smaller than the file size, so the transmission time or signal overhead required for broadcasting file request and transmission messages is ignored when calculating (2). After the file transmission message broadcasting, each node *i* simultaneously transmits file $f_{i^{*}}$ for all i∈[1:n].

It is worthwhile mentioning that the synchronization between nodes is required to be established in the above file request and transmission procedure. Such synchronization might be achievable for orthogonal frequency-division multiplexing (OFMD) systems if the propagation delay between nodes is within the cyclic prefix interval of OFMD systems. Additionally, it is assumed that codebooks are shared among nodes before communication or all the nodes use the same codebook for file delivery.

**Figure 1:**
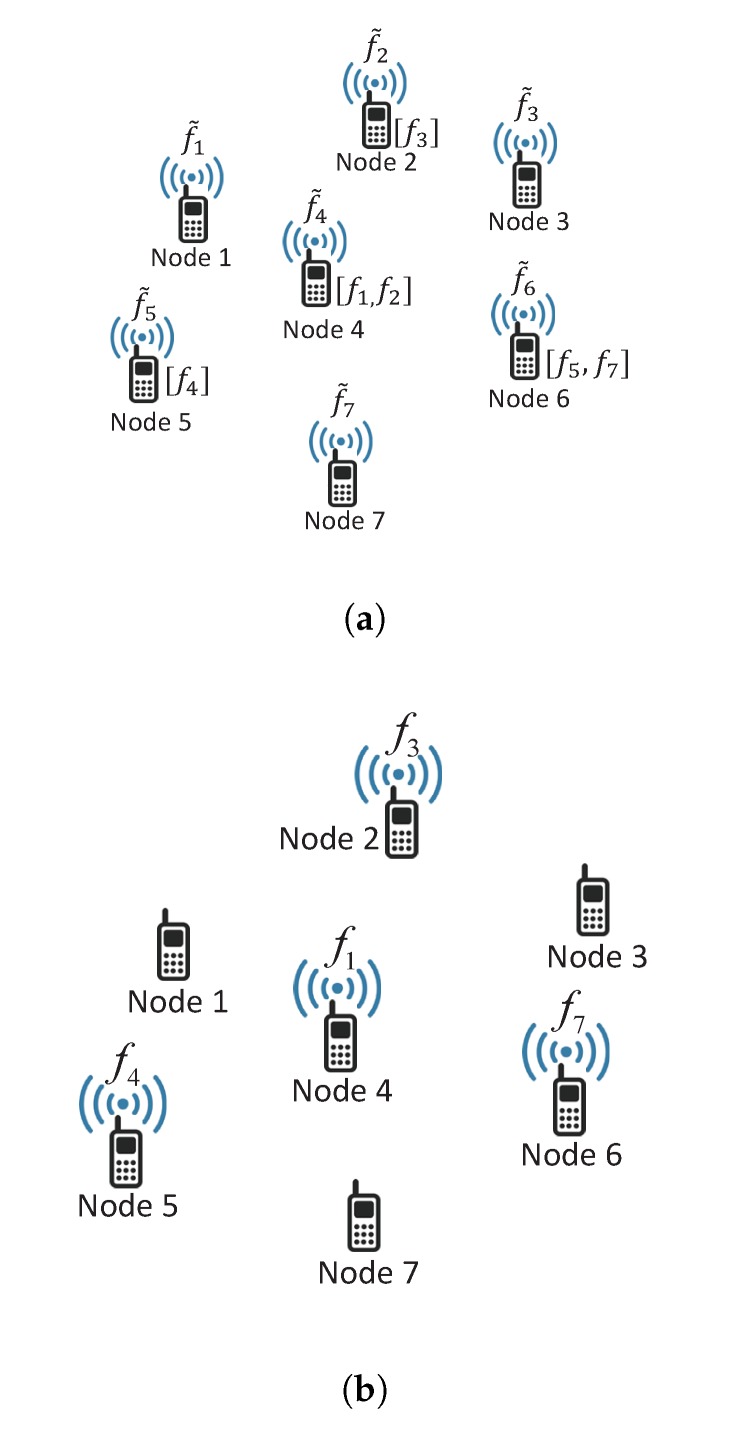
Example of file request message and file transmission. (**a**) file request message, (**b**) file transmission.

Figure 1 illustrates an example of file request message and the corresponding file transmission, where [·] in Figure 1a indicates a cached file at each node and it is assumed that $f_{i}\neq f_{j}$ for i≠j for simplicity (Note that $f_{i}$ can be equal to $f_{j}$ even if i≠j in the proposed scheme in general). In the example, nodes 1, 3, and 7 do not transmit any file since the y do not have any requested files in their cache memories, and node 4 transmits file $f_{1}$ since the distance from node 1 is closer than the distance from node 2 (and this also applies to node 6).

### 3.2. Step 2: Interference Cancellation at the Receiver Side

Suppose that each node transmits a requested file (or does not transmit) according to Step 1. For k∈[1:m], let $N_{\left[k\right]}\subseteq \left[1:n\right]$ denote the subset of nodes that transmits a file $f_{\left[k\right]}$. In addition, $N_{\left[0\right]}\subseteq \left[1:n\right]$ denotes the subset of nodes that does not transmit any file. From the file request message and file transmission message broadcasting in Step 1, the sets ${\left\{N_{\left[k\right]}\right\}}_{k\in [1:m]}$ are available at each node, i.e., the file indices for received signals are reported at each node as in [6,8]. Such file indices are needed to perform the first-stage IC stated in Section 3.2.1. Note that $N_{\left[k\right]}\cap N_{\left[l\right]}=Ø$ for k≠l and ${⋃}_{k=0}^{m}N_{\left[k\right]}=\left[1:n\right]$ from the definition. Denote the transmit signal associated with file $f_{\left[k\right]}$ by $x_{\left[k\right]}$, which is generated according to CN(0,P) and independent over different *k*. That is, all nodes in $N_{\left[k\right]}$ transmit the same signal $x_{\left[k\right]}$ to deliver file $f_{\left[k\right]}$. Then, from (1), the received signal of node *j* is represented by $y_{j}=\sum_{k=1}^{m}\sum_{i\in N_{\left[k\right]}}h_{ji}x_{\left[k\right]}+n_{j}$ from the fact that $x_{i}=x_{\left[k\right]}$ for all $i\in N_{\left[k\right]}$ and $x_{\left[0\right]}=0$ (no transmission). Define

(3) $$\begin{array}{c} g_{j[k]}:=\sum_{i\in N_{\left[k\right]}}h_{ji}=\sum_{i\in N_{\left[k\right]}}\frac{q_{ji}}{d_{ji}^{\gamma /2}} \end{array}$$

as the effective channel coefficient from the nodes in $N_{\left[k\right]}$ to node *j*, where $g_{j[k]}=0$ if $N_{\left[k\right]}=Ø$. Then, we have

(4) $$\begin{array}{c} y_{j}=\sum_{k=1}^{m}g_{j[k]}x_{\left[k\right]}+n_{j}. \end{array}$$

In the proposed scheme, node *j* first exploits its cached files $F_{j}$ for the two-stage IC as stated below.

#### 3.2.1. First-Stage Interference Cancellation

The first-stage IC utilizes cached files as side information, i.e., this strategy directly removes interference signals caused by the files in $F_{j}$ by subtracting $\sum_{k=1,f_{\left[k\right]}\in F_{j}}^{m}g_{j[k]}x_{\left[k\right]}$ from the received signal $y_{j}$. Then, after the first-stage IC, we have

(5) $$\begin{array}{cc} y_{j}^{\left(0\right)} & :=y_{j}-\sum_{k=1,f_{\left[k\right]}\in F_{j}}^{m}g_{j[k]}x_{\left[k\right]}\\ \; & =\sum_{k=1,f_{\left[k\right]}\in F\setminus F_{j}}^{m}g_{j[k]}x_{\left[k\right]}+n_{j}. \end{array}$$

Suppose that node *j* attempts to decode file $f_{\left[l\right]}$ from $y_{j}^{\left(0\right)}$ in (5), where $f_{\left[l\right]}\in F\setminus F_{j}$. Note that $f_{\left[l\right]}$ can be the desired file $f_{j}$. Then, the average SINR value for decoding $f_{\left[l\right]}$ from $y_{j}^{\left(0\right)}$, denoted by $\eta_{j[l]}^{\left(0\right)}$, is given by

(6) $$\begin{array}{c} \eta_{j[l]}^{\left(0\right)}=\frac{E[|g_{j[l]}{|}^{2}]P}{1+\left(\sum_{k=1,f_{\left[k\right]\in F\setminus (F_{j}\cup \left\{f_{\left[l\right]}\right\})}}^{m}E[|g_{j[k]}{|}^{2}]\right)P} \end{array}$$

from the fact that the average received desired signal power (caused by file $f_{\left[l\right]}$) is given by $E\left[|g_{j[l]}x_{\left[l\right]}{|}^{2}\right]=E[|g_{j[l]}{|}^{2}]P$ and that the average received interference power (caused by the files in $F\setminus (F_{j}\cup \left\{f_{\left[l\right]}\right\})$) is given by

(7) $$\begin{array}{cc} \; & E\left[|\sum_{k=1,f_{\left[k\right]}\in F\setminus \left(F_{j}\cup \left\{f_{\left[l\right]}\right\}\right)}^{m}g_{j[k]}x_{\left[k\right]}{|}^{2}\right]=\left(\sum_{k=1,f_{\left[k\right]}\in F\setminus \left(F_{j}\cup \left\{f_{\left[l\right]}\right\}\right)}^{m}E[|g_{j[k]}{|}^{2}]\right)P, \end{array}$$

since $x_{\left[k\right]}$ is independent over different *k* and channel coefficients. Here, $E[|g_{j[k]}{|}^{2}]$ is given by

(8) $$\begin{array}{cc} E[|g_{j[k]}{|}^{2}] & =E\left[|\sum_{i\in N_{\left[k\right]}}\frac{q_{ji}}{d_{ji}^{\gamma /2}}{|}^{2}\right]=\sum_{i\in N_{\left[k\right]}}d_{ji}^{-\gamma } \end{array}$$

from the fact that $q_{ji}$ is independent over different *i* whose variance is one. Therefore, up to the first-stage IC, node *j* is able to decode file $f_{\left[l\right]}$ from $y_{j}^{\left(0\right)}$ if

(9) $$\begin{array}{c} \log_{2}\left(1+\eta_{j[l]}^{\left(0\right)}\right)\geq R, \end{array}$$

i.e., $\eta_{j[l]}^{\left(0\right)}\geq 2^{R}-1$.

#### 3.2.2. Second-Stage Interference Cancellation

The second-stage IC is related to so-called interference decoding [9], in which each node firstly decodes a subset of interfering files and then cancels out their contributions before decoding the intended file. Hence, some strong interfering files can be removed, which results in an improved SINR for decoding the intended file and, as a result, enhancing the outage-based throughput performance in Section 2.2.

For the second-stage IC, node *j* first decodes a subset of undesired files and then eliminates their contributions before decoding the desired file. Denote $U_{j}\subseteq \left[1:m\right]$ as the set of cached file indices in $F_{j}$ and denote $V_{j}=\left[v_{j}\left(1\right),v_{j}\left(2\right)\cdots ,v_{j}\left(m-M\right)\right]\subseteq \left[1:m\right]$ as the set of ordered file indices of $\left[1:m\right]\setminus U_{j}$ satisfying $E[|g_{j[v_{j}\left(l\right)]}{|}^{2}]\geq E[|g_{j[v_{j}\left(l^{\prime }\right)]}{|}^{2}]$ for $l\leq l^{\prime }$, where $l,l^{\prime }\in \left[1:m-M\right]$. This results in $\eta_{j[v_{j}\left(l\right)]}^{\left(0\right)}\geq \eta_{j[v_{j}\left(l^{\prime }\right)]}^{\left(0\right)}$ if $l\leq l^{\prime }$. Consequently, node *j* cannot decode files $f_{\left[v_{j}\left(2\right)\right]},f_{\left[v_{j}\left(3\right)\right]},\cdots ,f_{\left[v_{j}\left(m-M\right)\right]}$ if it cannot decode file $f_{\left[v_{j}\left(1\right)\right]}$, i.e.,

(10) $$\begin{array}{c} \log_{2}\left(1+\eta_{j[v_{j}\left(1\right)]}^{\left(0\right)}\right)<R. \end{array}$$

Therefore, in the proposed successive IC, node *j* first attempts to decode $f_{\left[v_{j}\left(1\right)\right]}$ from $y_{j}^{\left(0\right)}$ in (5).

Suppose that $\eta_{j[v_{j}\left(1\right)]}^{\left(0\right)}\geq 2^{R}-1$ so that node *j* successfully decodes file $f_{\left[v_{j}\left(1\right)\right]}$. Then, by subtracting the interference signal caused by file $f_{\left[v_{j}\left(1\right)\right]}$ from $y_{j}^{\left(0\right)}$ in (5), we have

(11) $$\begin{array}{cc} y_{j}^{\left(1\right)} & =y_{j}^{\left(0\right)}-g_{j[v_{j}\left(1\right)]}x_{\left[v_{j}\left(1\right)\right]}\\ \; & =\sum_{k=1,f_{\left[k\right]}\in F\setminus \left(F_{j}\cup \left\{f_{\left[v_{j}\left(1\right)\right]}\right\}\right)}^{m}g_{j[k]}x_{\left[k\right]}+n_{j}. \end{array}$$

Similarly, node *j* cannot decode files $f_{\left[v_{j}\left(3\right)\right]},\cdots ,f_{\left[v_{j}\left(m-M\right)\right]}$ if it cannot decode file $f_{\left[v_{j}\left(2\right)\right]}$ from $y_{j}^{\left(1\right)}$. Therefore, node *j* now tries to decode $f_{\left[v_{j}\left(2\right)\right]}$ from $y_{j}^{\left(1\right)}$ in (11).

The second-stage IC sequentially performs the above procedure, i.e., successive IC with decoding order $v_{j}\left(1\right),v_{j}\left(2\right)\cdots ,v_{j}\left(m-M\right)$ until the desired file $f_{j}$ is decoded. If the desired file $f_{j}$ is not included in the set of decoded files, then an outage occurs for node *j*. Notice that the received signal of node *j* after successive IC of the decoded files $v_{j}\left(1\right),\cdots ,v_{j}\left(l-1\right)$ is given by

(12) $$\begin{array}{c} y_{j}^{\left(l-1\right)}=\sum_{k=1,f_{\left[k\right]}\in F\setminus \left(F_{j}\cup \left\{f_{\left[v_{j}\left(1\right)\right]},\cdots ,f_{\left[v_{j}\left(l-1\right)\right]}\right\}\right)}^{m}g_{j[k]}x_{\left[k\right]}+n_{j}, \end{array}$$

and the average SINR value for decoding file $v_{j}\left(l\right)$ from $y_{j}^{\left(l-1\right)}$ is given by

(13) $$\begin{array}{cc} \; & \eta_{j[v_{j}\left(l\right)]}^{\left(l-1\right)}=\frac{E[|g_{j[v_{j}\left(l\right)]}{|}^{2}]P}{1+(\sum_{k=1,f_{\left[k\right]\in F\setminus (F_{j}\cup \left\{f_{\left[v_{j}\left(1\right)\right]},\cdots ,f_{\left[v_{j}\left(l\right)\right]}\right\})}}^{m}E[|g_{j[k]}{|}^{2}])P}, \end{array}$$

where l∈[1:m-M]. By comparing (13) with (6), we can confirm the SINR improvement due to the second-stage IC.

The pseudocode of the second-stage IC for each node *j* is summarized in Algorithm 1.
**Algorithm 1:** Second-stage IC of the proposed scheme.
 1 **Initialization**: Set $F_{j}^{\mathrm{dec}}=Ø$ and construct $y_{j}^{\left(0\right)}$ and $V_{j}$ from the first-stage IC.

 2 **For**
l=1:m-M
 3 **If**
$\eta_{j[v_{j}\left(l\right)]}^{\left(l-1\right)}\geq 2^{R}-1$
 4  Decode $f_{\left[v_{j}\left(l\right)\right]}$ from $y_{j}^{\left(l-1\right)}$.
 5  Update $F_{j}^{\mathrm{dec}}\to F_{j}^{\mathrm{dec}}\cup \left\{f_{\left[v_{j}\left(l\right)\right]}\right\}$.
 6  Construct $y_{j}^{\left(l\right)}$ from $y_{j}^{\left(l-1\right)}$ by cancelling interference caused by $f_{\left[v_{j}\left(l\right)\right]}$.
 7  **If**
$f_{j}\in F_{j}^{\mathrm{dec}}$
 8   **Return** ‘no outage’ for node *j*.
 9  **End**
 10 **Else**
 11   **Return** ‘outage’ for node *j*.
 12 **End**
 13 **End**
 14 **Result**: ‘no outage’ or ‘outage’ for node *j*.


## 4. Simulation Results

In this section, we numerically evaluate the achievable outage-based throughput of the proposed scheme in (2).

### 4.1. Caching Placement

To maximize the hit probability, we apply the recently established caching placement in [7]. For completeness, we briefly introduce the optimal caching placement proposed in [7] as below:

**Remark** **2** *(Caching placement optimization).* *In [7], the optimal probabilistic caching placement that maximizes the hit probability has been proposed under a homogeneous Poisson Point Process (PPP) with node density λ. Let $\rho_{\left[k\right]}$ denote the caching probability of file k at each node, where $\sum_{k=1}^{m}\rho_{\left[k\right]}=M$. Then, the optimal caching probability that maximizes the hit probability within the radius τ is represented by $\rho_{\left[k\right]}^{⋆}=\min\left\{\max\left\{\rho_{\left[k\right]}\left(\mu^{⋆}\right),0\right\},1\right\}$, where*

(14) $$\begin{array}{c} \rho_{\left[k\right]}\left(\mu \right)=-\frac{\omega \left\{\frac{\mu }{p_{r}\left(f_{\left[k\right]}\right)}\exp\left(1+\pi \lambda \tau^{2}\right)\right\}}{\pi \lambda \tau^{2}}+\frac{1}{\pi \lambda \tau^{2}}+1. \end{array}$$

*Here, ω denotes the Lambert W function and $\mu^{⋆}$ can be obtained by the bisection search method, satisfying $\sum_{k=1}^{m}\rho_{\left[k\right]}^{⋆}=M$.*

In addition, as a simple baseline caching placement, we also consider a random caching placement in which each node caches *M* different files uniformly at random among *m* files in the library.

### 4.2. Simulation Environment

In simulations, we set a=10, n=100, and γ=4. Recall that $a^{2}$, *n*, and γ denote the network area, the number of nodes, and the path-loss exponent, respectively. We assume that the short-term fading $q_{ji}$ follows CN(0,1), i.e., Rayleigh fading, and the file popularity distribution follows a Zipf distribution with the Zipf exponent ν [22]. That is,

(15) $$\begin{array}{c} p_{r}\left(f_{\left[k\right]}\right)=\frac{k^{-\nu }}{\sum_{l=1}^{m}l^{-\nu }}. \end{array}$$

The simulations were implemented in Matlab. Since we assumed static network topology, the path-loss component between nodes is fixed during the entire time block, i.e., $\frac{1}{d_{ji}^{\gamma /2}}$, but the short-term fading component $q_{ji}\left[t\right]$ varies over time slots. We further average out the outage-based throughput performance by conducting simulation for a large enough number of time blocks, i.e., averaging for random network topology. The parameters *R* and δ are numerically optimized to maximize the outage-based throughput in (2) for each simulation. Furthermore, we assume an 8-bit uniform analog-to-digital converter (ADC) at each node such that the maximum signal strength of each ADC is set as the average received signal power transmitted at a unit distance. Therefore, if $d_{ji}<1$, the received signal power is saturated with the same value as when $d_{ji}=1$. For the same reason, if $d_{ji}>4$, the received signal power will be less than the lowest signal level of each ADC, and signal detection is impossible for this case. Table 1 summarizes the parameters used in simulations.

To demonstrate the performance of the proposed scheme, we compare it with three benchmark schemes. As a baseline scheme, we first consider the conventional D2D file delivery without any IC, which is denoted as ‘no IC’. In [6,8], IC by utilizing cached files as side information has been proposed, which is the second benchmark scheme, denoted as ‘IC with cached files’. It is worthwhile mentioning that IC or successive IC by interference decoding of undesired files has been widely adopted in the literature, for examples, see [9,10]. In addition, cooperative transmission gain from multiple nodes sending the same file has been utilized in [4,8] for caching networks, which can be adopted in IC or successive IC by interference decoding. Hence, the last benchmark scheme combines the above two techniques, which is simply denoted as ‘IC by interference decoding’.

### 4.3. Numerical Results and Discussions

In this subsection, we present our numerical results and discuss the performance of the proposed scheme with respect to the parameters such as Zipf exponent, caching capability, and library size. We further discuss the effect of channel estimation error on the performance of the proposed scheme.

#### 4.3.1. Throughput Comparison with Respect to the Zipf Exponent

**Figure 2:**
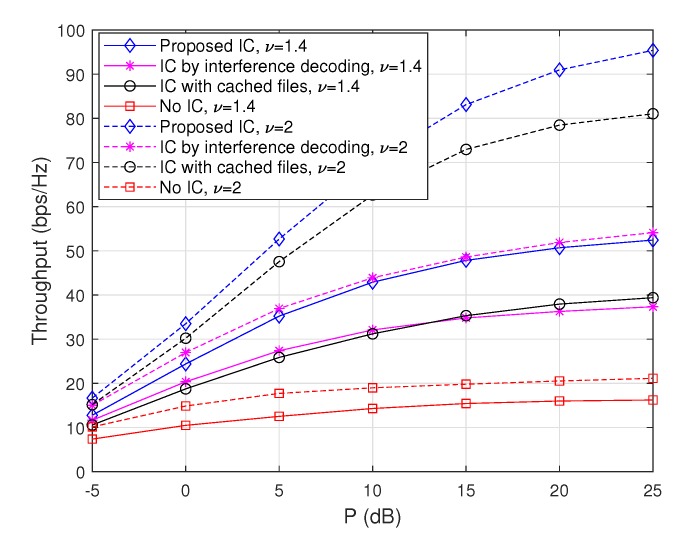
Throughputs as a function of *P* when m=50 and M=10, where the max-hit caching placement is used.

**Figure 3:**
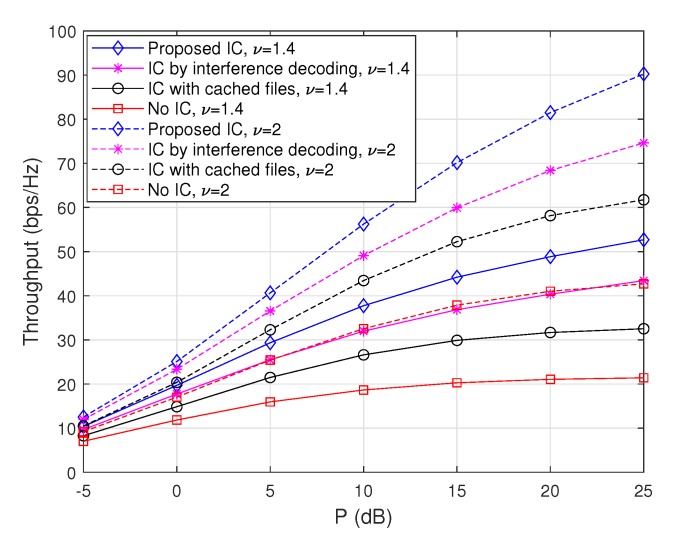
Throughputs as a function of *P* when m=50 and M=10, where the random caching placement is used.

**Figure 4:**
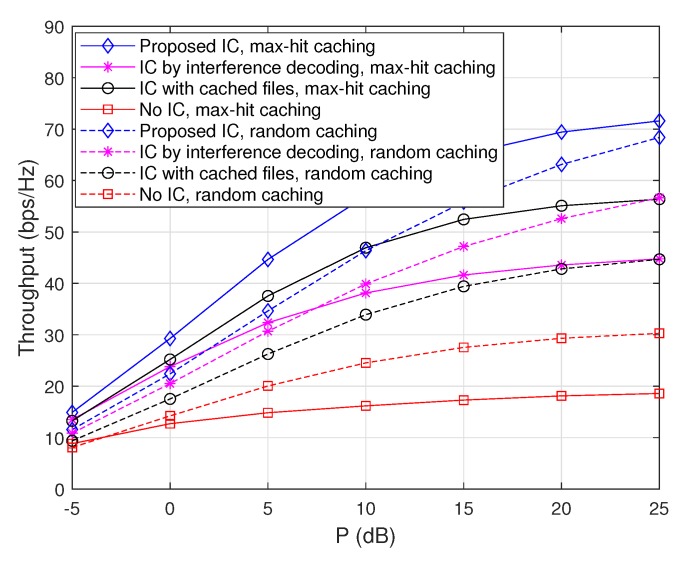
Throughputs as a function of *P* when m=50, M=10, and ν=1.7.

To evaluate the performance tendency of the proposed scheme with respect to the Zipf exponent ν, we plot achievable throughputs when ν=1.4 and ν=2 for the max-hit caching placement in Figure 2 and for the random caching placement in Figure 3. Note that the cases when ν=1.7 are depicted in Figure 4. From these figures, it is observed that throughputs increase with ν regardless of the choice of caching placement strategies or interference cancellation schemes. This is because the gain from cooperative D2D file delivery increases with ν, since the probability that each node sends the same popular file increases with ν. Furthermore, in the case of the max-hit caching placement, throughputs of ‘IC with cached files’ increase significantly with ν compared to those of ‘IC by interference decoding’, because the amount of interfering signals that each node can remove by using cached files increases as the popularity of files is concentrated on a certain subset of files. On the other hand, in a similar vein, it is observed that throughput improvement attained from ‘IC by interference decoding’ becomes dominant in the case of random caching placement compared to max-hit caching placement, since non-optimal caching placement reduces the gain of the cache-enabled IC. More importantly, the ‘Proposed IC’ scheme provides a synergistic throughput improvement compared to the cases of ‘IC with cached files’ and ‘IC by interference decoding’ for a wide range of ν.

#### 4.3.2. Throughput Comparison with Respect to Caching Capability

**Figure 5:**
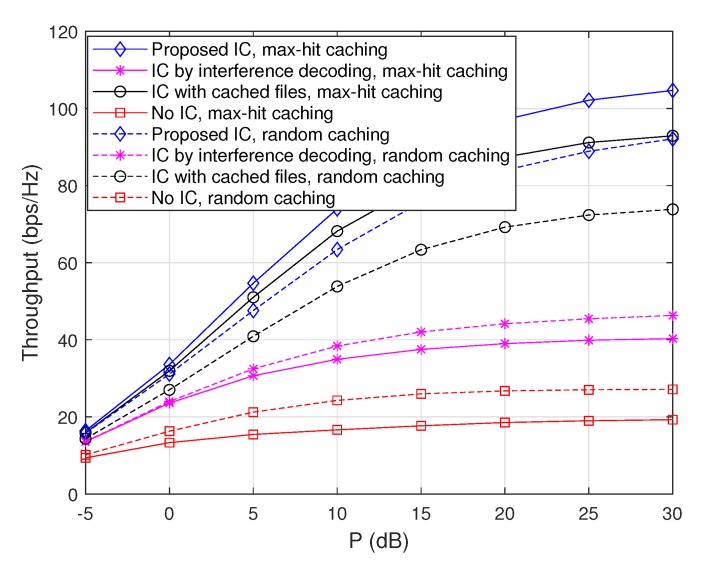
Throughputs as a function of *P* when m=20, M=10, and ν=1.7.

To evaluate the performance tendency with respect to caching capability, we plot achievable throughputs for m=50 and m=20 in Figure 4 and Figure 5, respectively, when M=10 and ν=1.7. In particular, Figure 4 plots achievable throughputs of the considered schemes as a function of *P* when m=50, M=10, and v=1.7. As expected, the max-hit caching placement achieves an improved throughput compared to the random caching placement for ‘IC with cached files’ and ‘Proposed IC’, but it achieves worse throughputs for ‘IC by interference decoding’ and ‘No IC’ due to the fact that cached files cannot be used as side information while the total amount of interfering signals at each node increases because of the maximized hit probability due to the optimal caching placement. Furthermore, again, the ‘Proposed IC’ scheme provides a significant throughput improvement compared to the cases of ‘IC with cached files’, ‘IC by interference decoding’, and ‘no IC’, and this is true independent of caching placement strategies.

For instance, the proposed scheme provides 26%, 59.3%, and 283.1% throughput improvements compared to ‘IC with cached files’, ‘IC by interference decoding’, and ‘no IC’ for the max-hit caching placement when P=20 dB, respectively, and it also provides 47.5%, 20%, 115.3% improvements compared to ‘IC with cached files’, ‘IC by interference decoding’, and ‘no IC’ for the random caching placement when P=20 dB, respectively.

As seen in Figure 5, similar tendency can be observed when m=20, M=10, and ν=1.7, except the fact that the gain from ‘IC with cached files’ relatively increases. This is because the frequency with which cached files are used as side information increases as the entire file size *m* is reduced with fixed *M*.

#### 4.3.3. Throughput comparison with Respect to the Library Size

**Figure 6:**
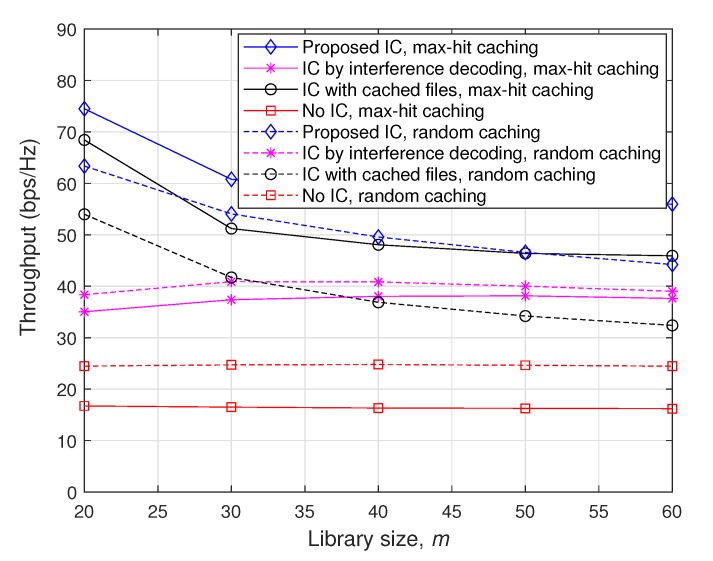
Throughputs as a function of *m* when M=10, P=10 dB, and ν=1.7.

Figure 6 plots achievable throughputs with respect to the library size *m* when M=10, P=10 dB, and ν=1.7. As seen in the figure, the throughput enhancement attained by the ‘Proposed IC’ scheme becomes larger as the cache memory size *M* is relatively smaller than the entire file size *m*, as discussed in Section 4.3.2. Furthermore, it is observed that the performance gap between ‘Proposed IC’ and ‘IC with cached files’ increases with *m*. Note that as *m* increases, the optimized maximum distance for D2D file delivery becomes larger so that there might be strong interfering nodes between D2D file delivery pairs. For such cases, ‘IC by interference decoding’, which is also implemented in the proposed scheme, can efficiently remove such strong interfering signals and thus play an important role in improving throughputs, especially for large *m*.

#### 4.3.4. Impacts of Imperfect Channel Estimation at The receiver Side

**Figure 7:**
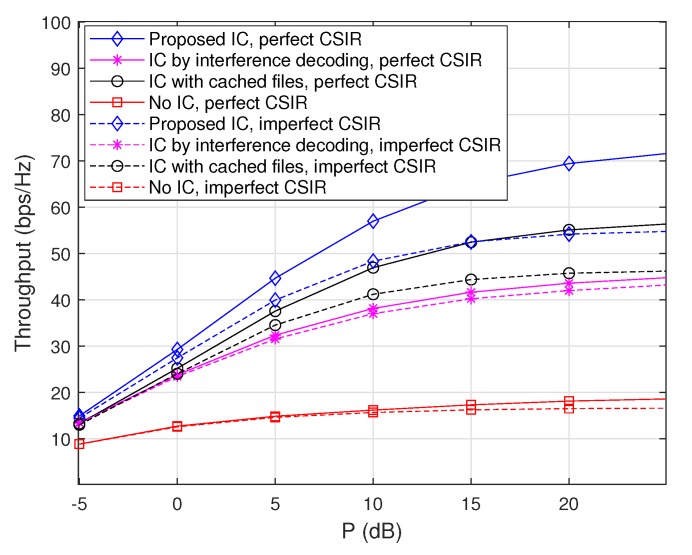
Throughputs as a function of *P* when m=50, M=10, and ν=1.7 with and without channel estimation error at the receiver side, where the max-hit caching placement is used.

**Figure 8:**
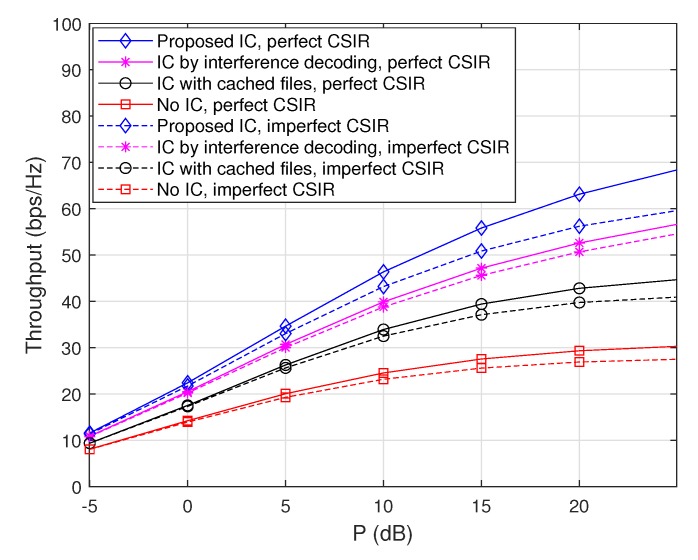
Throughputs as a function of *P* when m=50, M=10, and ν=1.7 with and without channel estimation error at the receiver side, where the random caching placement is used.

Throughout the paper, we have assumed perfect channel state information at the receiver side (CSIR), but channel estimation error may exist in practice. Therefore, here we examine the impacts of channel estimation error on throughputs. Specifically, the mean squared error (MSE) of the channel estimation is assumed to be given by 0.05 for all the channel coefficients, i.e., the short-term fading $q_{ji}$ is given by $q_{ji}={\bar{q}}_{ji}+{\tilde{q}}_{ji}$, where the estimated channel coefficient ${\bar{q}}_{ji}$ follows CN(0,1) while the channel estimation error ${\tilde{q}}_{ji}$ follows CN(0,0.05), for all i,j∈[1:n]. Figure 7 and Figure 8 plot throughputs as a function of *P* when m=50, M=10, and ν=1.7 with and without channel estimation error at the receiver side, where the max-hit caching placement and the random caching placement are used, respectively. The results demonstrate that the overall performance tendency in the presence of channel estimation error is similar to that with perfect CSIR in both the caching placement strategies. Furthermore, the ‘Proposed IC’ scheme still provides a synergistic throughput improvement over the other schemes, even in the presence of channel estimation error.

## 5. Concluding Remarks

In this paper, we developed a new file delivery scheme for wireless D2D caching networks, consisting of cooperative transmission at the transmitter side and two-stage IC at the receiver side. Specifically, in the proposed two-stage IC, each node first removes interfering signals by using cached files as side information and then performs successive IC in which the decoding order is determined by the received signal power. Numerical simulations demonstrated that the proposed IC scheme significantly outperforms the conventional scheme regardless of the choice of caching policies, and the performance gain due to the proposed scheme increases as the cache memory size *M* becomes relatively smaller compared to the entire file size *m*.

In practice, we might need to consider dynamics of network topologies and file popularity distributions. For such a case, the procedure of file request and selection of sender nodes needs to be modified depending on the node mobility. In addition, although most of the caching research in the literature, including our work, assumed a static file popularity distribution; in practice, some new files will be appeared and old files or non-popular files will be disappeared over time, which results in a time-varying file popularity distribution [23]. Therefore, file placement in each cache memory should be periodically updated. For such a case, signaling overhead or communication cost for such a periodic update should be reflected in overall caching gain.

## Figures and Tables

**Table 1:** Simulation parameters.

Parameter	Assumption
Network area, a×a	100
Number of nodes, *n*	100
Path-loss exponent, γ	4
Channel model	Rayleigh fading
Channel estimation	Perfect at the receiver side, unknown to the transmitter side
File popularity distribution	Zipf distribution
Analog to digital converter (ADC) resolution	8 bits
